# Countermovement Jump Force–Time Curve Analysis between Strength-Matched Male and Female Soccer Players

**DOI:** 10.3390/ijerph19063352

**Published:** 2022-03-12

**Authors:** Christopher Thomas, Paul A. Jones, Thomas Dos’Santos

**Affiliations:** 1 Directorate of Psychology and Sport, University of Salford, Salford M6 6PU, UK; p.a.jones@salford.ac.uk; 2 Department of Sport and Exercise Sciences, Musculoskeletal Science and Sports Medicine Research Centre, Manchester Metropolitan University, Manchester M15 6BH, UK; t.dossantos@mmu.ac.uk

**Keywords:** gender differences, waveform analysis, isometric mid-thigh pull, statistical parametric mapping

## Abstract

The purpose of this study was to compare countermovement jump force–time measures between strength-matched male and female soccer players. Males (*n* = 11) and females (*n* = 11) were strength-matched via isometric mid-thigh pull testing, whereby peak force values were normalised to body mass. Subjects performed three maximal-effort countermovement jumps (CMJs) on a force platform from which a range of kinetic and kinematic variables were calculated via forward dynamics. Thereafter, differences in gross measures were examined via independent *t*-tests, while differences in force–, power–, velocity–, and displacement–time curves throughout the entire CMJ were analysed using statistical parametric mapping (SPM). Jump height, reactive strength index modified, propulsion mean force, propulsion impulse, and propulsion mean velocity were all greater for males (d = 1.50 to 3.07). Relative force– and velocity–time curves were greater for males at 86–93% (latter half of the concentric phase) and 85–100% (latter half of the concentric phase) of normalized movement time, respectively. Time to take-off, braking phase time, braking mean velocity and impulse, propulsion phase time and centre of mass displacement were similar between males and females (d = −0.23 to 0.97). This research demonstrates the strength of SPM to identify changes between entire force-time curves. Continued development and the use of SPM analysis could present the opportunity for a refined comparison of strength-matched male and female CMJ performance with the analysis of entire force–time curves.

## 1. Introduction

Vertical jump tasks, such as the countermovement jump (CMJ) have been frequently researched and are commonly used to assess and monitor the capacity of the lower body to produce impulse in team and individual sports [1,2,3,4]. Furthermore, the CMJ induces minimum fatigue, requires minimum familiarization, and is highly reliable [5], demonstrating its usefulness in elite sport as part of ongoing performance and monitoring assessments. Typical variables derived from CMJ testing using force platforms including jump height, peak force, peak power, peak velocity, time to take-off, and rate of force development have been used to monitor training outcomes, fatigue and overtraining potential, and readiness to train [1,6,7]. Numerous attempts have been made to identify differences in performance measures between males and females [3,4,8,9,10], yet it is questionable whether a lack of control or consideration of physical capacity (e.g., strength) potentially confounds much of this research [11,12].

Several investigations have sought to determine differences in CMJ performance between males and females [3,4,8,9,10]. Prior evidence suggests that males demonstrate greater jump height [9], relative peak concentric force [10], eccentric impulse [3], propulsive impulse [13], and peak power than females. Specifically, McMahon et al. [9] found males jumped higher by displacing their centre of mass more during ground contact, but within a similar time, achieving a greater velocity throughout most of the concentric phase. Yet, inconsistences are evident in previous analyses and definitions of variables. For example, eccentric calculations have included the unweighting phase [3], while impulse calculations failed to deduct bodyweight [3,13]. Likewise, the omission of measures such as velocity [3,13] and centre of mass displacement [3] may miss CMJ strategy changes but does however provide clear outcome-based performance measures. Furthermore, a lack of matching other variables (e.g., strength) may potentially confound much of this research. Indeed, recent findings demonstrated that matching males and females for strength may negate some of the previously observed differences in CMJ performance [3,4]. Rice et al. [3] found male–female differences were not a determining factor in CMJ performance when relative strength-matched in the isometric back squat. Additionally, previous research demonstrates resistance training interventions elicit similar levels of improvement in muscular strength as well as neural adaptations in males and females [14]. Thus, variables that are modifiable through training, such as strength, may likely explain previous conclusions attributed to gender/sex.

A strength-matched participant approach has scarcely been implemented [11], but provides a strong research design to elucidate actual differences between males and females versus modifiable differences attributed to training history or, in this specific example, muscular strength. The research to date has tended to focus on discrete point analyses which only provide an insight into a small part of CMJ performance as a large portion of data across the waveform are left unexamined. An alternative approach is to explore differences in CMJ force–time curves [6,15] to inform practitioners of potential temporal and movement strategy changes across the entire CMJ movement. Indeed, previous studies were able to identify differences in time-normalized relative force, velocity, and centre of mass displacement [9], and relative power [3,4] between males and females. Thus, strength-matched subjects and full waveform analysis may provide a greater insight into the mechanical differences between male and female CMJ performances. Such advantages are that one-dimension analysis uses Random Field Theory [16] instead of performing separate inferential tests at each time point, reducing Type I error. Furthermore, one-dimensional analysis allows for non-directed hypotheses on the portion of the curve where changes may occur [16]. The main aim of this study was to investigate the differences in CMJ phase characteristics by comparing force–, velocity–, and displacement–time curves in strength-matched male and female soccer players. A secondary purpose of this study was to examine several gross measure values between subjects.

## 2. Methods

### 2.1. Experimental Approach to the Problem

A cross-sectional between-subjects comparative design was used to compare CMJ gross and temporal characteristics between strength-matched male and female soccer players. All subjects visited the Human Performance Laboratory for a single testing session. Subjects attended the laboratory for a familiarization session <7 days prior to data collection, in-spite of all subjects being familiar with the tests performed as part of their normal training and monitoring regime.

### 2.2. Subjects

Eleven female (age = 20.73 ± 0.79 years; height = 1.66 ± 0.09 m; body mass = 54.70 ± 6.34 kg) and eleven male (age = 21.00 ± 1.90 years; height = 1.78 ± 0.05 m; body mass = 76.82 ± 7.53 kg) collegiate soccer players participated in this study. Subjects were all of similar resistance training background (>2-years, 2–3 ×/week). All subjects were free of injury at the time of testing. Written informed consent was provided prior to testing and the study was pre-approved by the university institutional review board, and it conformed to the World Medical Association’s Declaration of Helsinki.

### 2.3. Procedures

Following a rest day, all subjects attended a testing session in a fed and hydrated state, similar to their normal training routine. Standing height (Stadiometer; Seca, Birmingham, UK) and body mass were assessed (Seca Digital Scales, Model 707) while in bare feet, and measured to the nearest 0.1 kg and 0.1 cm, respectively. Countermovement jump testing was performed first, followed by isometric strength testing separated by 10 min of rest. Prior to testing, subjects performed a standardized warm-up, as they would prior to any normal training session. Additionally, standardized warm-up trials of CMJ and isometric strength were performed.

### 2.4. Countermovement Jump

Following a brief dynamic warm-up (including sub-maximal CMJ attempts), subjects performed three CMJs (interspersed with one minute of rest) to a self-selected depth. Subjects were instructed to perform the CMJs as fast and as high as possible, whilst keeping their arms akimbo. Any CMJs that were performed with the inclusion of arm swing or tucking of the legs during the flight phase of the jumps were omitted, and additional CMJs were performed after one minute of rest. All vertical ground reaction force (GRF) CMJ data were recorded at 1000 Hz via a Kistler type 9286AA force platform and Bioware 5.11 software (Kistler Instruments Inc., Amherst, NY, USA). The raw vertical force–time data for each CMJ trial were exported and analysed using a customized Microsoft Excel spreadsheet (version 2016, Microsoft Corp., Redmond, WA, USA).

**Figure 1:**
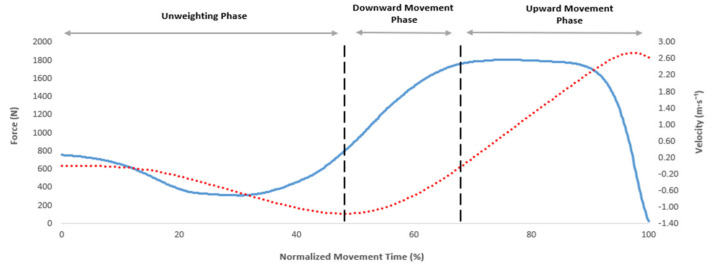
Countermovement jump phase interpretation based on force-time (blue solid line) and velocity-time (red dotted line) curve data.

Based on recent recommendations [17], centre of mass velocity was calculated by dividing net vertical force by body mass and then integrating the product using the trapezoid rule. Thereafter, instantaneous COM displacement was calculated by numerically integrating the velocity–time record using the trapezoid rule [18]. Bodyweight and the start of CMJ were identified as the instant when vertical force was reduced by a threshold equal to 5 times the standard deviation (SD) of BW (calculated in the weighing phase) [18]. The CMJ phases were identified using the terminology explained recently [17]. Briefly, the unweighting phase was defined as occurring between the onset of downward movement and the instant of peak negative velocity, the downward movement phase was defined as occurring between the end of the unweighing phase and when COM velocity equalled zero, and the upward movement phase was deemed to have started when velocity became positive (exceeded 0.01 m·s^−1^) and finished at take-off [18] (Figure 1).

Braking and propulsion phase time were defined as the time taken to perform the braking and propulsion phases, respectively. Braking and propulsion mean force and mean velocity were defined as the average of the values attained during the braking and propulsion phases, respectively. Impulse was calculated during both the braking and propulsion phases of the jump as the area under the net force–time curve (excluding body weight) using the trapezoid rule [19]. All kinetic data were divided by body mass to allow for normalized comparison of these data between males and females. Similarly, countermovement and propulsion displacement were expressed as a percentage of standing centre of mass height, calculated as 55 and 57% of standing height for females and males, respectively [20], to enable fairer between-group comparison. The vertical velocity of the COM at take-off was used to calculate jump height [21]. Jump height was divided by time to take-off to calculate reactive strength index modified (RSImod) [2]. Full waveform analyses of CMJ trials were conducted by normalizing each subject’s force–, velocity–, and displacement–time curves to 101 nodes (0–100% of movement time).

### 2.5. Isometric Mid-Thigh Pull

Following CMJ trials, subjects were provided with a rest period of approximately 10 min before the isometric mid-thigh pull (IMTP) test. For the IMTP, previously described procedures were used [22]. Briefly, using a portable IMTP rig (Fitness Technologies, Perth, Australia), an immovable cold-rolled steel bar was positioned at a height that replicated the start of the second pull phase of the clean for each subject, with the bar fixed above the force platform to accommodate subjects of different sizes and proportions to optimize the individual athlete’s pulling position. This posture resulted in knee and hip angles of 125.3 ± 6.6° and 143.7 ± 8.4°, respectively [22]. Three warm-up trials (50, 75, and 90%) of the subject’s perceived maximum effort were performed, each separated by 1 min of rest. Once body position was stabilized, the subjects were given a countdown of “3, 2, 1, Pull”, with the instruction to pull against the bar “and push the feet directly into the ground as fast and hard as possible”. All subjects were given strong verbal encouragement during each trial. Subjects performed 3 maximal-effort IMTP trials interspersed with 2 min of rest between trials.

Vertical GRF data for the IMTP was collected using a portable force platform sampling at 600 Hz (400 Series Performance Force Plate, Fitness Technology, Adelaide, Australia) interfaced with a laptop computer and specialist software (Ballistic Measurement System, Fitness Technology, Adelaide, Australia). Raw, unfiltered, force–time data were exported for subsequent analysis in a bespoke Microsoft Excel spreadsheet (version 2016, Microsoft Corp., Redmond, WA, USA). The maximum forces recorded from the force–time curve during the IMTP trials were reported as PF and subsequently ratio scaled (force/body mass [N·kg^−1^]). All force data represented net force (maximum force—body weight). The onset of force production was defined as the point when force exceeded 5 times the standard deviation (SD) of BW (calculated in the weighing phase) [23]. The best performance of the three trials was used for further analysis.

### 2.6. Statistical Analysis

Within-session reliability of CMJ measures was determined via a two-way random-effects model intraclass correlation coefficient (ICC). The ICC values were interpreted as poor (<0.50), moderate (0.50–0.75), good (0.75–0.90), and excellent (>0.90) [24]. Absolute between-trial variability of each gross variable was calculated using the coefficient of variation (sample mean/sample standard deviation) expressed as a percentage (%CV). Normality of data was confirmed by Shapiro–Wilk statistic and Q–Q plot analysis. All data satisfied parametric assumptions, except RSImod, braking mean force, and propulsion mean force. Raw values in each parametric variable derived for females and males were compared using independent *t*-tests, whereas RSImod, braking mean force, and propulsion mean force were compared via the Mann–Whitney U test. *t*-tests and ICCs were performed using SPSS software (version 24; SPSS Inc., Chicago, IL, USA) with the alpha level set at *p* ≤ 0.003. Effect sizes were calculated using the Cohen’s d method [25] and interpreted as trivial (≤0.19), small (0.20–0.49), moderate (0.50–0.79), or large (≥0.80), and 95% confidence intervals were calculated for ICC, CV% and effect sizes. Differences in force–, velocity–, and displacement–time curves were determined via independent *t*-tests in open-source SPM software located at http://www.spm1d.org/, accessed on 20 October 2021 [16]. The scalar output SPM{t*} was calculated separately at each individual data point and is referred to as a statistical parametric map indicating the magnitude of the difference between data. Where the scalar output statistic crossed the critical threshold ({F} and {*t*}), the null hypothesis was rejected. The threshold value for SPM{t*} was set at *p* < 0.05.

## 3. Results

Descriptive statistics revealed males were significantly (large effect) taller (*p* = 0.001, d = 1.56) and heavier (*p* < 0.001, d = 2.95), but there was no significant (trivial effect) between-group difference in age (*p* = 0.946, d = 0.14), with male subjects being slightly older.

Within-session-reliability of all IMTP and CMJ variables was good to excellent (ICC = 0.69–0.98), with coefficients of variation between 1.23 and 11.74%.

Males jumped significantly higher than females and demonstrated significantly greater RSImod values despite time to take-off being similar between groups (Table 1). Within a similar time to take-off, males displayed significantly greater propulsion mean force and braking and propulsion impulse. Furthermore, males demonstrated significantly greater braking and propulsion mean velocity than females. Time to take-off, braking and propulsion phase times, and countermovement and propulsion COM displacements were similar between males and females, as was braking mean force (Table 1).

**Figure 2:**
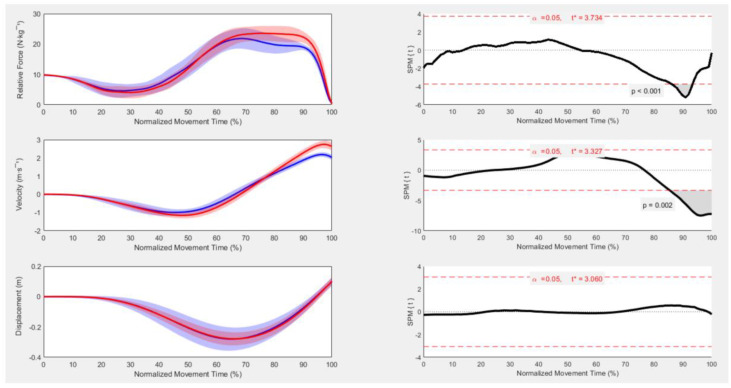
Mean ± SD vertical GRF waveforms (**left panels**) and SPM{t*} output statistics for force-time (**upper**), velocity-time (**middle**) and displacement-time (**bottom**) curves. Where the SPM{t*} curve exceeds the critical threshold (dotted line), this area is shaded and a statistically significant relationship is present at those nodes with *p* values provided for each supra-threshold cluster. Males = red; females = blue.

SPM analysis showed that relative force was greater for males between 86 and 93% of the movement, which corresponded to the latter half of the concentric phase of the jump (Figure 2). Velocity was greater for males between 85 and 100% of the movement (Figure 2), which also corresponded to the latter half of the concentric phase of the jump. There were no differences in COM displacement between males and females across the full movement (Figure 2).

## 4. Discussion

The aim of this study was to investigate the differences in CMJ phase characteristics by comparing force–, velocity–, and displacement–time curves in strength-matched male and female soccer players. A secondary purpose of this study was to examine several gross measure values between subjects. The current study found that in strength-matched males and females, males jumped higher than females due to a larger propulsion net impulse, resulting in a higher propulsion velocity based on the impulse–momentum relationship. Velocity at take-off dictated jump height. Our results demonstrate that males achieved a greater jump height within a similar time to take-off and COM displacement as females. The current investigation was unique in that strength-matching the male and female subjects was performed to remove maximal strength (relative to body mass) as a confounding variable in determining differences between the CMJ phase characteristics of male and female soccer players. It should be noted that these results apply to a multi-joint isometric task (isometric mid-thigh pull) and may not be applicable to other knee and hip angles or dynamic tasks. Future investigations may want to examine strength-matching between males and females in both isometric and dynamic multi-joint tasks.

The finding that relative propulsion mean force, as well as the force–time signature recorded throughout the jump, demonstrated differences between males and females is in line with similar work reporting relative peak values [10], but in contrast to many other studies [3,9,13]. This finding may partly be explained by the fact that structural differences in the muscle’s elastic properties, such as muscle pennation angle and motor unit activation, have previously shown to be different between males and females, contributing to force production and power transference. Specifically, greater pennation angles of the vastus lateralis and gastrocnemius lateralis in males than females are shown to correlate with greater jump height and peak power in the CMJ [26]. Likewise, males can activate more motor units, resulting in greater force development due to a larger muscle cross-sectional area and greater vastus lateralis pennation angle [27]. Yet, previous research has shown that resistance training interventions elicit similar levels of improvement in muscular strength as well as neural adaptations in males and females [14]. It may be that CMJ performance relies on impulse, as it is the only way to accelerate the body, according to the impulse–momentum theorem. Impulse is a combination of force and time, so greater force means greater impulse, with males demonstrating superior braking and propulsion impulse compared to females in the current study. Comparison of the findings with those of other studies confirms that males demonstrate greater impulse values than females [3,9,13]. Advantageous stretch-shortening cycle activation might explain the greater braking and propulsion impulse achieved by males, resulting in higher jump heights. Although muscle pennation angles and cross-sectional area measurements were not taken into consideration in the current study, body mass was significantly greater in males. Future studies should continue to investigate dynamic athletic tasks between males and females, statistically controlling for strength differences as well as muscle characteristics, where possible.

Prior studies have shown that males adopt greater (stiffer) leg stiffness strategies than females during various hopping frequencies, with a stiffer strategy related to shorter movement times [28] and larger forces [28,29]. Time to take-off between males and females was statistically similar in the present study, as in previous work [8,10]. Consequently, the males tested in the current study achieved an impulse that was larger in force rather than longer in duration. Males achieved higher RSImod values than the females (Table 1) due to greater jump heights rather than reduced time to take-off. Jump height is the effect of the impulse applied to the ground from start to take-off. Thus, jumping higher and with a shorter time to take-off should be of importance for sports such as soccer, as this would produce the preferential tall- and thin-style of impulse [9]. This point also highlights that RSImod should not be looked at in isolation, but to consider the components, while considering the jump strategy, to help direct training priorities from any testing and monitoring program.

The current study found that males jumped ~0.15 m (+43%) higher than females, as previously reported, and values ranged between 0.09 and 0.15 m [3,10,26]. Despite this similarity, the absolute values of the sample in the current study were below the aforementioned studies [3,8,10], but in-line with those previously reported in female (RSImod: 0.28 ± 0.06; jump height: 0.24 ± 0.03 m) and male (RSImod: 0.44 ± 0.09; jump height: 0.35 ± 0.06 m) Division I NCAA soccer players [30]. It could be possible that both groups tested in the present study would benefit from increasing maximal force production, given the relationship between maximal strength and vertical jump height [31]. Improvements in maximal force production capabilities has also shown to improve eccentric force– and power–time characteristics of the CMJ [7,32,33]. Several lines of evidence suggest that resistance training results in an increase in muscle cross-sectional area and muscle-tendon stiffness [34,35], which has been shown to be reduced in females [36], and contributes to improving maximal force and power capabilities [37,38]. Rice et al. [3] found between-group differences in CMJ kinetics in strength-matched males and females, potentially due to differential jump strategies employed (e.g., squat depth). In the current study, matching relative strength and accounting for squat depth did not show any differences in braking and propulsion times (and resultant time to take-off), or countermovement and propulsion COM displacement. Vertical jump performance has previously shown to be affected by the velocity and depth of the countermovement [39]; thus, as no differences were observed in countermovement depth in the current study, the superior jump performance of males can also be attributed to higher mean velocity during the braking phase, resulting in a larger braking impulse, which is expected to allow the development of higher force outputs at the onset and throughout the propulsive phase [40], subsequently increasing propulsion impulse.

This study contributes to our understanding of full waveform analysis in the CMJ [3,4,9] by providing an analysis of strength-matched male and female time-normalized movement patterns using SPM. The current study found that relative force was greater for males between 86 and 93% of the movement, while velocity was greater between 85 and 100% of the movement. Examining full waveform analysis allowed us to detect differences throughout the entire force trace, and could provide a detailed analysis of “how” the jump was achieved, which may be concealed when conducting gross variable analysis. Thus, gross analysis may be useful to measure a desired performance outcome of a jump (i.e., propulsion mean velocity or jump height), whereas SPM can provide deeper analytical information on the movement strategy by presenting information relative to the entire movement. In the current study, performing gross and full waveform analysis shows the potential of SPM to detect greater sensitivity to force data, suggesting that SPM could prove advantageous in extending our knowledge on differences in CMJ performance between males and females.

A limitation of the study is that the small sample size (total, *n* = 22, group, *n* = 11) did not allow us to address position differences within the sport. Previous research has indicated that players of different positions within the same sport may exhibit different strength–power characteristics [41], possibly due to technical proficiency based on movement demands of the respective positions. However, subjects in the current study were of a homogeneous training history and current training status, while all being outfield players, with no goalkeepers included in testing. Furthermore, all subjects conducted CMJs regularly within their training and monitoring program and demonstrated high consistency in their three jump performances, which lends support to the validity of the current findings. Soccer players were recruited for the current study, but it is unknown whether the findings can be extrapolated to other sports (i.e., rugby, cricket, hockey, tennis). This study did not evaluate leg muscle activity in isometric or dynamic tasks. A recent study by Nimphius et al. [12] indicated no difference in muscle activity between strength-matched males and females. It should be noted that the IMTP peak force values reported in this study are lower than those reported in an excellent review of the literature by Brady et al. [42] Rather than either the male group being relatively weak, or the female group being relatively strong, it appears both subject groups within the current study were relatively weaker compared to male and female subject values from previous works. Thus, the findings of the current study should be interpreted with the athlete population, methodology, and method of calculating variables in mind when comparing normative data across research.

Despite its limitations, this study certainly adds to our understanding of the CMJ force–time curve analysis between males and females when removing strength as a confounding factor. The present study raises the possibility that females may benefit from using a more compliant strategy during the CMJ to increase jump height, while still aiming to perform the jump in as short a time as possible. This combination should enable a greater concentric impulse to be achieved—importantly, force dominant and then time dominant—to meet the time constraints of soccer-specific tasks. The greater relative forces demonstrated by males may suggest that training to produce CMJ propulsion forces over shallower ranges (less hip, knee, and ankle extension) may be beneficial to improve CMJ. It is important to bear in mind these changes may reduce jump height acutely, but should rebound with improved rapid force-production capabilities. Thus, both maximal strength and ballistic resistance training could be major factors, causing positive adaptations to the neuromuscular factors that affect CMJ performance, prescribing the necessary exercises, loads, and rep-set configurations based on their individual needs.

## 5. Conclusions

Controlling for covariates such as maximal strength allows researchers to more accurately identify the factors or variables which underpin performance differences between males and females with respect to the CMJ. This study aimed to investigate differences in CMJ phase characteristics by comparing force–, velocity–, and displacement–time curves and several gross measures in strength-matched male and female soccer players. Importantly, our findings demonstrated that male soccer players achieved a greater jump height within a similar time to take-off and COM displacement as female soccer players, primarily through differences in force, impulse, and velocity. Thus, given maximal strength was controlled for, the importance of the soccer and any prior multi-sport training backgrounds’ influence on jump strategy is an important consideration. It could well be that, in the current study, female players had less history of soccer and sport training practice, potentially leading to differences in jump strategy, compared to male soccer players. Therefore, jump strategy may have a greater influence on CMJ performance than differences between sexes. This highlights the need for further research regarding CMJ performance in both males and females across a variety of sports. Nonetheless, our findings have important implications for monitoring and assessing CMJ performance and, more notably, training interventions to elicit changes in CMJ performance.

## Figures and Tables

**Table 1:** Comparison of gross countermovement jump variables.

	Female (*n* = 11)		Male (*n* = 11)		*p*	d (95% CI)	ES	ICC (95% CI)	%CV (95% CI)
Variable	Mean	SD	Mean	SD					
Isometric mid-thigh pull peak force (N·kg^−1^)	23.49	5.30	24.94	5.60	0.541	0.21 (−0.64 to 1.07)	Unclear	0.96 (0.93 to 0.98)	1.23 (1.02 to 1.55)
Mass (kg)	54.70	6.34	76.82	7.53	<0.001 *	2.95 (2.09 to 3.80)	Large		
RSImod	0.29	0.08	0.46	0.09	0.001 *	1.92 (1.06 to 2.78)	Large	0.90 (0.80 to 0.96)	11.74 (9.45 to 16.01)
Jump height (m)	0.21	0.03	0.36	0.06	<0.001 *	3.07 (2.21 to 3.93)	Large	0.99 (0.97 to 0.99)	4.14 (3.35 to 5.57)
Time to take-off (s)	0.784	0.188	0.788	0.142	0.949	0.07 (−0.79 to 0.93)	Unclear	0.80 (0.62 to 0.91)	9.72 (7.84 to 13.21)
Braking phase time (s)	0.169	0.053	0.156	0.026	0.920	0.05 (−0.81 to 0.91)	Unclear	0.82 (0.65 to 0.92)	10.49 (8.46 to 14.28)
Propulsion phase time(s)	0.264	0.059	0.249	0.033	0.530	−0.23 (−1.10 to 0.63)	Unclear	0.91 (0.81 to 0.96)	5.92 (4.79 to 8.00)
Countermovement COM displacement (%)	27.74	7.70	29.66	5.34	0.445	0.36 (−0.50 to 1.22)	Unclear	0.90 (0.81 to 0.96)	8.87 (7.16 to 12.05)
Propulsion COM displacement (%)	36.92	8.14	39.86	6.27	0.339	0.45 (−0.40 to 1.31)	Unclear	0.94 (0.87 to 0.97)	5.21 (4.22 to 7.03)
Braking mean force (N·kg^−1^)	17.36	2.18	18.30	1.96	0.224	0.43 (−0.43 to 1.29)	Unclear	0.86 (0.73 to 0.94)	5.12 (4.14 to 6.91)
Propulsion mean force (N·kg^−1^)	17.86	1.85	20.48	1.47	0.002 *	1.50 (0.64 to 2.36)	Large	0.93 (0.85 to 0.97)	3.37 (2.73 to 4.53)
Braking impulse (N·kg^−1^·s)	1.10	0.17	1.28	0.22	0.041	0.86 (0.01 to 1.71)	Large	0.90 (0.80 to 0.95)	6.99 (5.66 to 9.47)
Propulsion impulse (N·kg^−1^·s)	2.04	0.15	2.64	0.23	<0.001 *	3.06 (2.20 to 3.91)	Large	0.99 (0.97 to 0.99)	1.97 (1.60 to 2.64)
Braking mean velocity (m·s^−1^)	0.71	0.10	0.82	0.13	0.026	0.97 (0.11 to 1.83)	Large	0.93 (0.85 to 0.97)	5.37 (4.35 to 7.25)
Propulsion mean velocity (m·s^−1^)	1.32	0.10	1.62	0.14	<0.001 *	2.31 (1.45 to 3.17)	Large	0.97 (0.94 to 0.99)	2.61 (2.12 to 2.51)

Notes: ES = effect size; COM = centre of mass; RSImod = reactive strength index modified; ES = effect size; ICC = intraclass correlation coefficient; CV = coefficient of variation; SD = standard deviation. * denotes statistical significance at *p* < 0.003.

## Data Availability

All relevant data are within the manuscript.
